# ST-YOLO: a deep learning based intelligent identification model for salt tolerance of wild rice seedlings

**DOI:** 10.3389/fpls.2025.1595386

**Published:** 2025-06-02

**Authors:** Qiong Yao, Pan Pan, Xiaoming Zheng, Guomin Zhou, Jianhua Zhang

**Affiliations:** ^1^ Agricultural Information Institute, Chinese Academy of Agricultural Sciences, Beijing, China; ^2^ College of Agriculture, Henan University, Kaifeng, China; ^3^ National Nanfan Research Institute, Chinese Academy of Agricultural Sciences, Sanya, China; ^4^ Institute of Crop Sciences, Chinese Academy of Agricultural Sciences, Beijing, China; ^5^ Nanjing Institute of Agricultural Mechanization, Ministry of Agriculture and Rural Affairs, Nanjing, China; ^6^ National Agricultural Science Data Center, Beijing, China; ^7^ Institute of Western Agriculture, Chinese Academy of Agricultural Sciences, Changji, Xinjiang, China

**Keywords:** wild rice, salt resistance grade, intelligent evaluation identification, deep learning, seedling

## Abstract

**Background:**

In response to the limited models for salt tolerance detection in wild rice, the subtle leaf features, and the difficulty in capturing salt stress characteristics, resulting in low recognition and detection rates and accuracy, a deep learning-based ST-YOLO wild rice seedling salt tolerance phenotype evaluation and identification model is proposed.

**Method:**

In order to improve accuracy and achieve model lightweighting, a multi branch structure DBB (Diverse Branch Block) is used to replace the convolutional layers in the C2f module, and a reparameterization module C2f DBB is proposed to replace some C2f modules. Diversified feature extraction paths are introduced to enhance the ability of feature extraction; Introducing CAFM (Context Aware Feature Modulation) convolution and attention fusion modules into the backbone network to enhance feature representation capabilities while improving the fusion of features at various scales; Design a more flexible and effective spatial pyramid pooling layer using deformable convolution and spatial information enhancement modules to improve the model’s ability to represent target features and detection accuracy.

**Results:**

The experimental results show that the improved algorithm improves the average precision by 2.7% compared with the original network; the accuracy rate improves by 3.5%; and the recall rate improves by 4.9%.

**Conclusion:**

The experimental results show that the improved model significantly improves in precision compared with the current mainstream model, and the model evaluates the salt tolerance level of wild rice varieties, and screens out a total of 2 varieties that are extremely salt tolerant and 7 varieties that are salt tolerant, which meets the real-time requirements, and has a certain reference value for the practical application.

## 1 Introduction

With the abnormal changes in the Earth’s environment and irrational anthropogenic irrigation, the area of saline land is increasing globally. Studies have shown that land salinity is one of the biggest constraints to further stable development of rice production (Tian et al., 2020). Rice is a salt sensitive crop, and salt stress is one of the most important reasons affecting the high yield of rice (Lu et al., 2025). Therefore, the creation of new salt tolerant germplasm of rice through genetic improvement will be helpful for the development and utilization of saline rice cropping areas. Direct cultivation of common rice in saline land has the problems of low yield and poor quality (Quan et al., 2017). Therefore the development of new salt tolerant rice varieties and the mining of salt tolerant genes are crucial. Wild rice is a very valuable germplasm resource without artificial selection and rich in genetic polymorphism (Song et al., 2005). After a long period of natural selection, wild rice has formed a rich variety of mutation types and therefore has many excellent traits that rice does not have. Rice has also lost many excellent genes during its evolutionary process (Zheng et al., 2017).

Wild rice has strong salt tolerance and is capable of growing and reproducing in high salt environments, providing an important genetic resource for genetic improvement of rice (Shahzad et al., 2022). Wang et al. (Wang et al., 2017) identified LOC_Os03g61750 (OsTRM13) as a candidate methyltransferase for Am-modified rice.OsTRM13 transcript levels were significantly increased by salt stress and ABA treatment, and it was found that OsTrm13 The OsTRM13 protein was mainly located in the nucleus, and OsTRM13 overexpression plants showed better salt stress tolerance. Meng et al. (Meng et al., 2024) constructed a set of chromosome segment substitution lines (CSSLs) using wild rice as the donor parent and the cultivated rice Nipponbare (Nip) as the circulating parent, and selected a CSSL strain CSSL118 containing both alleles. CSSL118 showed a higher level of tolerance to salt stress compared with the parental Nip, and CSSL118 showed a higher level of tolerance than the parental Nip, and CSSL118 showed a higher level of tolerance than the parental Nip. CSSL118 showed comprehensive salt tolerance and higher yield than the parental Nip. Ge et al. (Ge et al., 2024) encoded an elite allele of LEA12OR, a late embryogenesis-enriched protein (LEA) in wild rice Oryza rufipogon Griff. and applied the LEA12OR allele to current rice through molecular breeding and genome editing. According to the wild rice seedling salt tolerance evaluation criteria, the leaf death rate needs to be calculated, and most of the current manual observation and assessment is used to derive the results, which suffers from inefficiency and errors. Therefore, efficient and accurate intelligent identification of salt tolerance phenotypes in wild rice is of great significance for the screening of salt tolerant germplasm resources in rice.

The evaluation and identification of salt tolerant germplasm resources based on deep learning has gradually received more attention and provided new perspectives and methods for the evaluation and identification of germplasm resources (Pardeep et al., 2024). Therefore, intelligent identification of salt tolerance in wild rice is of great significance for rice. Yao et al. (Yao et al., 2021) used 1D-CNN-LSTM technology to predict the electrical signals under long-term stress with different NaCl concentrations, and designed the NaCl stress concentration discrimination model (SCDM) and salt tolerance classification model (STCM), in which the accuracy of SCDM in distinguishing between NaCl stress concentrations was increased to 88% and 83%, and the accuracy of STCM in distinguishing salt tolerant and salt sensitive varieties reached 92.36%, which revealed the quantitative relationship between the electrical signals of wheat under different NaCl stress concentrations, salt tolerance and time-dependence of wheat, but there were problems such as high imbalance, insufficient quantity and low germination rate, which made it difficult to find more accurate quantitative relationships. Ma et al. (Ma et al., 2022) used Raman spectrometry to obtain molecular information of rice varieties and combined the Python deep learning and data visualization and analysis methods to establish an identification model for salinity-tolerant rice varieties, with an identification rate of 89.36%. In summary, the accuracy still needs to be improved, the model has not achieved lightweight, and the computational speed does not meet the requirements of practical applications.

Taking wild rice as the research object, this paper proposes an intelligent identification and evaluation model for salt tolerance of wild rice based on deep learning ST-YOLO network, and the Diverse Branch Block branch block (DBB) module is added to the backbone layer, replacing the Conv in Bottleneck in C2f, to improve the accuracy of the model. Meanwhile, the CAFM (Context Aware Feature Modulation) module is placed after backbone and before detect to enhance the extraction ability of global and local features, and improve the model’s characterization ability and detection accuracy of the target features. The ST-YOLO model is effectively enhanced in feature extraction and representation ability. Thus, it enhances the ability to accurately assess the salt tolerance of wild rice, which provides powerful support for wild rice salt tolerance research and breeding, and is of great significance for the screening of rice salt tolerant germplasm resources.

## 2 Materials and methods

### 2.1 Experimental methods

The experimental materials for this study were wild rice 1-254, all of which were obtained from the Institute of Crop Science, Chinese Academy of Agricultural Sciences. Test samples: wild rice seeds with full grains were selected and 30 seeds were randomly counted in each group by counting equipment or by hand.

Breaking seed dormancy: 30 seeds of wild rice were counted and placed in the oven, and then treated with high temperature from 45°C to 50°C for 72 h to break seed dormancy.

Constant temperature seed soaking and germination: After disinfection (1% NaClO, 25 min) seeds were placed in a climatic incubator for seed soaking under the conditions of ‘28°C, 48 h, and light shielding’. After soaking, the seeds were covered with double gauze and appropriate amount of distilled water was added, and then the petri dishes were placed in the climatic incubator for germination under the conditions of ‘fn °C, 48 h, and shadingon

**Figure 1:**
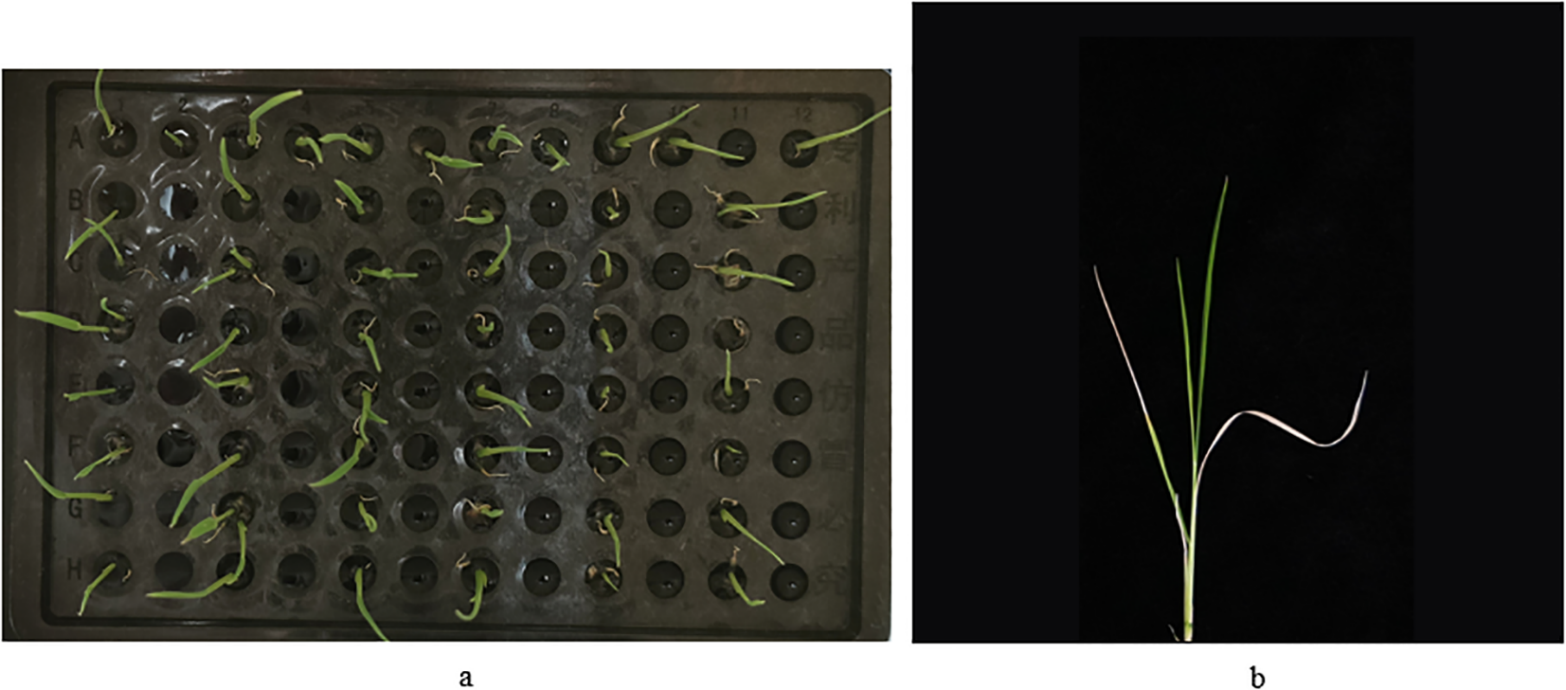
Process of salt treatment during seedling stage of wild rice. **(a)** Wild rice seed germination stage. **(b)** Photographs taken after recovery from salt treatment.

Hydroponics to two-leaf-one-heart stage: 20 seeds with good germination status were selected to be cultivated in Yoshida rice culture medium on a hydroponics box (plastic, 96 holes, pore diameter 6.3 mm, overall length 127 mm, width 87 mm, height 114 mm) under the conditions of ‘30°C, 12 h of light, 12000 Lx of light intensity’. Eight seeds were placed in one column, one variety in each column (**Figure 1a**). After 14 days of hydroponics, the seeds were grown to two leaves and one heart, and then subjected to salt stress.

Salt treatment: it was placed in Yoshida rice culture medium supplemented with 10t NaCl for incubation for 7 days. Then, they were placed in Yoshida rice culture medium for recovery culture and incubated for 7 days.

Data statistics: survival rates were counted on day 8 and photographs were taken (**Figure 1b**). There were 254 varieties of wild rice, and each variety was replicated with 8 plants. Image acquisition was performed on each salt treated wild rice seedling, and a total of 2032 images were collected. The image acquisition method was vertical shooting, in which the wild rice was individually placed on top of a black background cloth, and the device was photographed at a distance of 42.5 cm from it. The acquisition device was equipped with a rear 50 megapixels + 2 megapixels camera, set to 2x digital zoom, and the image format was JPG, and the photographs were all of high quality and full colors.

### 2.2 The construction of the dataset

**Figure 2:**
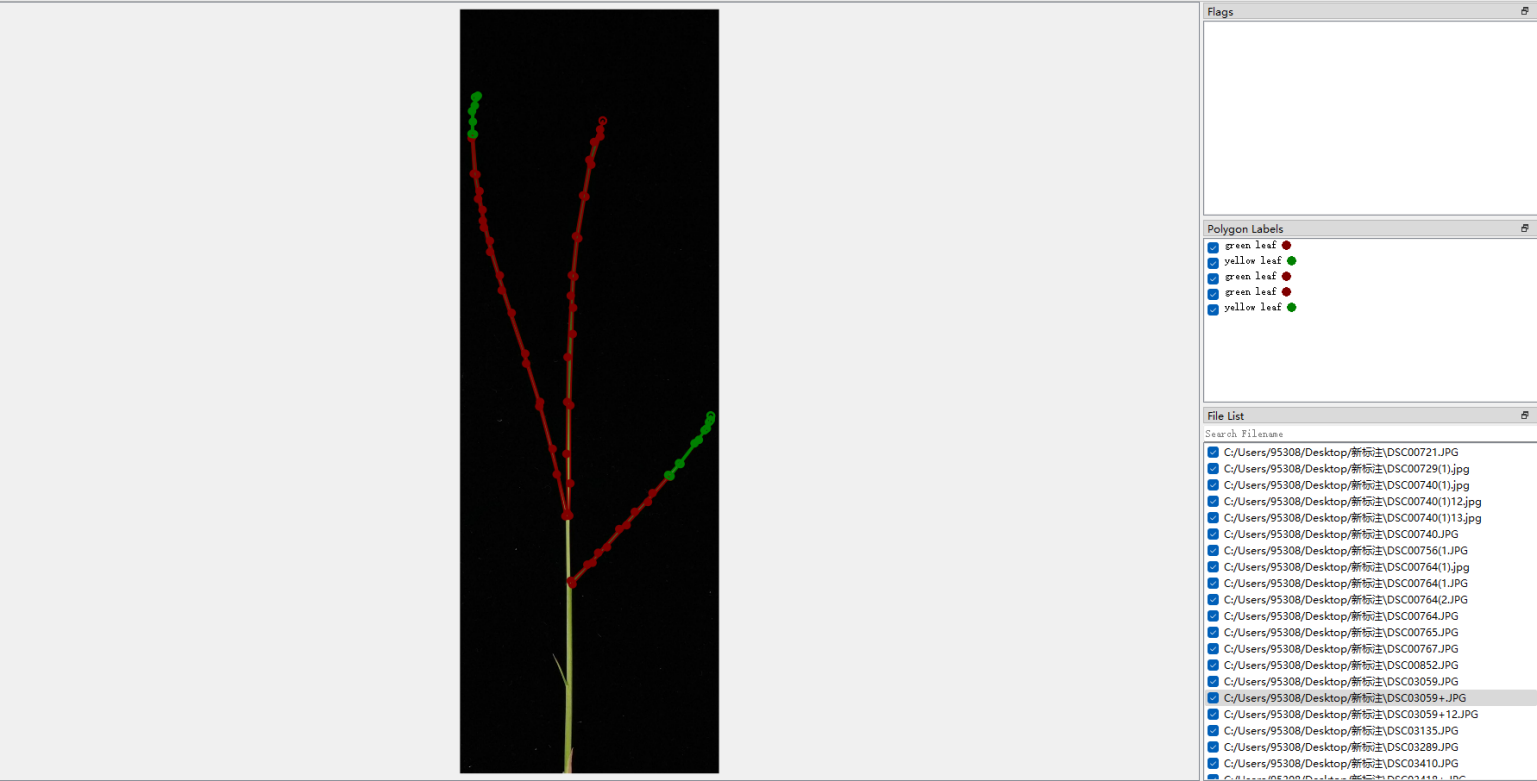
Labeling of the degree of salt damage to wild rice.

The wild rice leaves in the captured images were annotated using Labelme software as shown in **Figure 2**. A JSON file is generated for each annotated image containing information such as image size, label name, etc. The leaves in each image are annotated by Labelme software. The dataset is divided according to stratified sampling, with a total of 2032, and the division ratio is training set: validation set: testing set=7:2:1, which corresponds to the number of captured images as 1422, 406, and 204, respectively.

### 2.3 Identification model for phenotypic evaluation of salt tolerance in wild rice seedlings

#### 2.3.1 ST-YOLO network model

**Figure 3:**
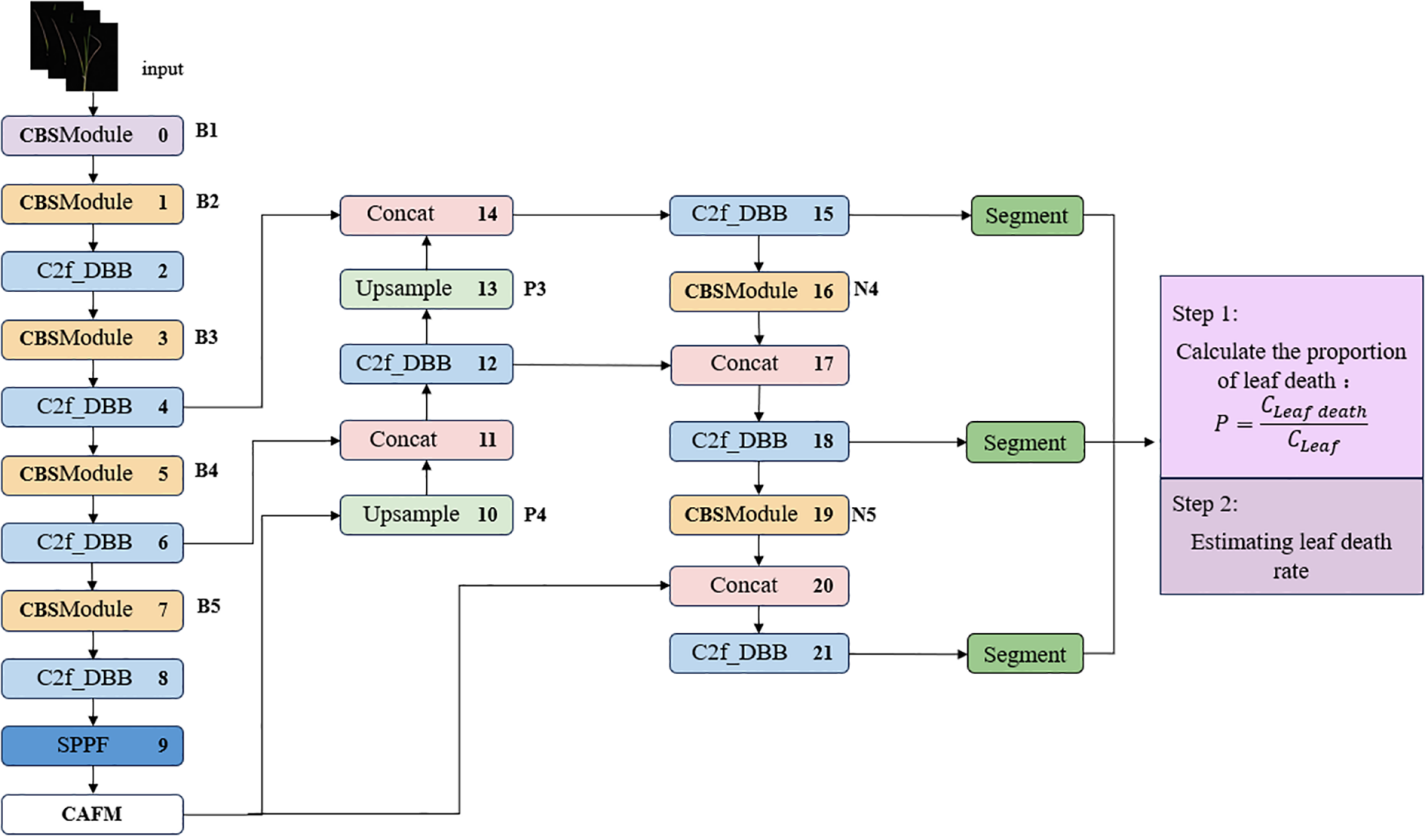
ST-YOLO technology roadmap.

In the context of salt stress, the alterations in leaf characteristics are minimal and challenging to accurately discern. The leaves of varying varieties exhibit diverse morphologies, overlap, and interspersion, in addition to variability in size, thereby complicating the process of leaf feature extraction. The manual detection of leaf death rate is a process that is difficult to quantify, which in turn leads to limited accuracy in calculating and analyzing the leaf death rate. This, in turn, adversely affects the accuracy of the salt tolerance detection results. The development of a deep learning-based system for the identification of salt tolerance in wild rice leaves involves the optimization of feature extraction capabilities and functionality to enhance the capture of image key features, thereby ensuring a more accurate and efficient model structure. The network architecture is depicted in **Figure 3**.

The ST-YOLO network structure is improved based on YOLOv8-Seg (Rejin and Sambath, 2024). The backbone of the network is the underlying architecture, which consists of convolutional layers, C2f module and spatial pyramid pooling module. The combination of multiple convolutional layers and residual blocks endows the network with powerful learning capability and extremely high detection accuracy, which can extract rich feature information from the input image. The C2f-DBB module is employed to extract feature information from the input image by replacing the convolution of the bottleneck part with the DBB module and adopting parallel data processing. This enables the capture of multi-dimensional features, which significantly improves the expressive ability of the network without a significant impact on the inference time. The CAFM module is incorporated at the end of the Backbone, and subsequently, the DBB mechanism is added to the C2f module in front of each detection head to enhance it into a C2f-DBB structure. With regard to the processing of loss during classification, YOLOv8-Seg employs Varifocal Loss, a technique that has been demonstrated to facilitate the network’s focus on the distribution of the adjacent regions of the target location. This, in turn, has been shown to result in enhanced target localization, along with improved accuracy and performance in target detection.

#### 2.3.2 YOLOv8-Seg

The YOLOv8 Seg developed by Ultralytics represents the latest progress in the YOLO series (Terven et al., 2023). Its architecture mainly consists of three core components: Backbone, Neck, and Head, which have been optimized and designed specifically for segmentation tasks.

We have adopted an optimized CSP backbone network. This backbone network has the ability to efficiently extract multi-scale features and extract rich details and semantic information from input images. The Neck section significantly enhances the interaction between features at different levels by integrating the feature pyramid network with the path aggregation network. This interaction enables the model to more accurately capture the boundary information of instances, resulting in higher accuracy in locating instances. The segmentation header combines pixel by pixel classification with instance discrimination. Pixel by pixel classification is responsible for determining the category to which each pixel belongs, while instance discrimination focuses on distinguishing different instances within the same category and accurately dividing the target. By randomly cropping, rotating, flipping, and other operations on the training data, the diversity of the data is increased, allowing the model to learn more different characteristics and patterns, and enhancing its ability to adapt to complex scenarios.

#### 2.3.3 The convolutional and attention fusion module

**Figure 4:**
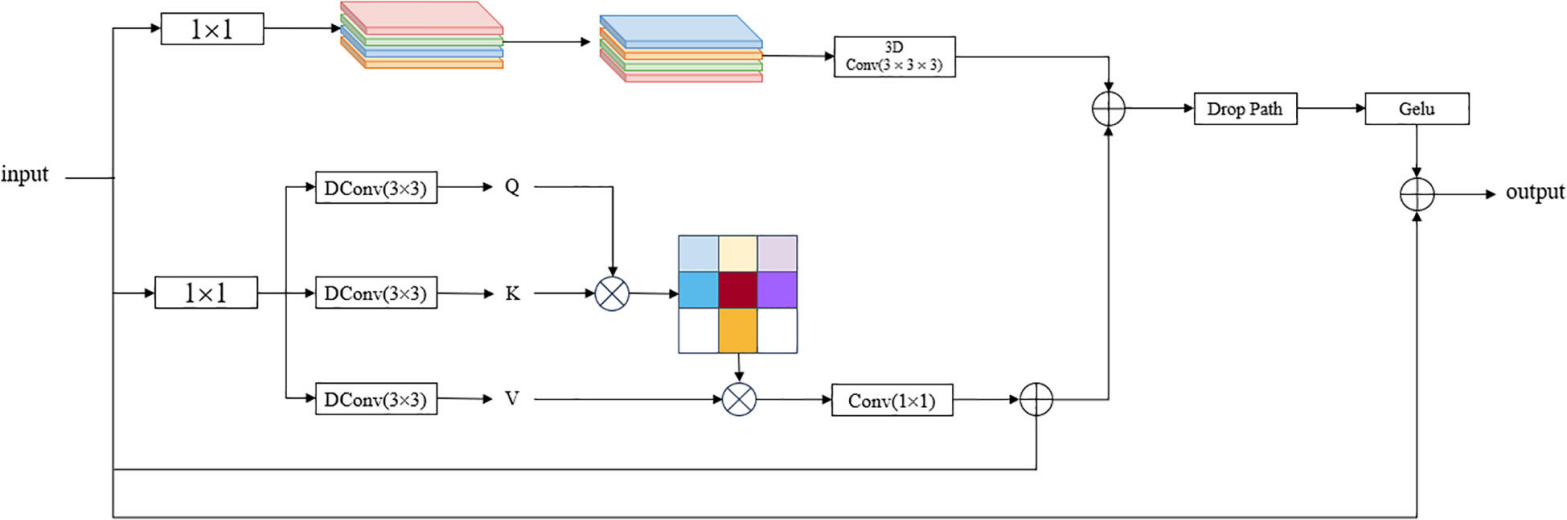
CAFM structure diagram.

The Convolutional and Attention Fusion Module (CAFM) (Hu et al., 2024) is a module that combines convolutional operations and attention mechanisms, aiming to improve the deep learning model’s attention to important features and learning ability. CAFM is introduced in YOLOv8-Seg to enhance the extraction of global and local features by placing it after backbone and before segment to effectively improve the performance of small target detection, and the enhanced feature representation enables the model to automatically learn and reinforce important features, which improves the model’s ability to understand and utilize the data. For PCB small target detection, due to the small size of the target, CAFM can extract features at different scales by designing a multi-scale feed-forward network, which in turn improves the detection performance for small targets, and the structure of CAFM is shown in **Figure 4**.

#### 2.3.4 Integration of DBB modules into the backbone layer

**Figure 5:**
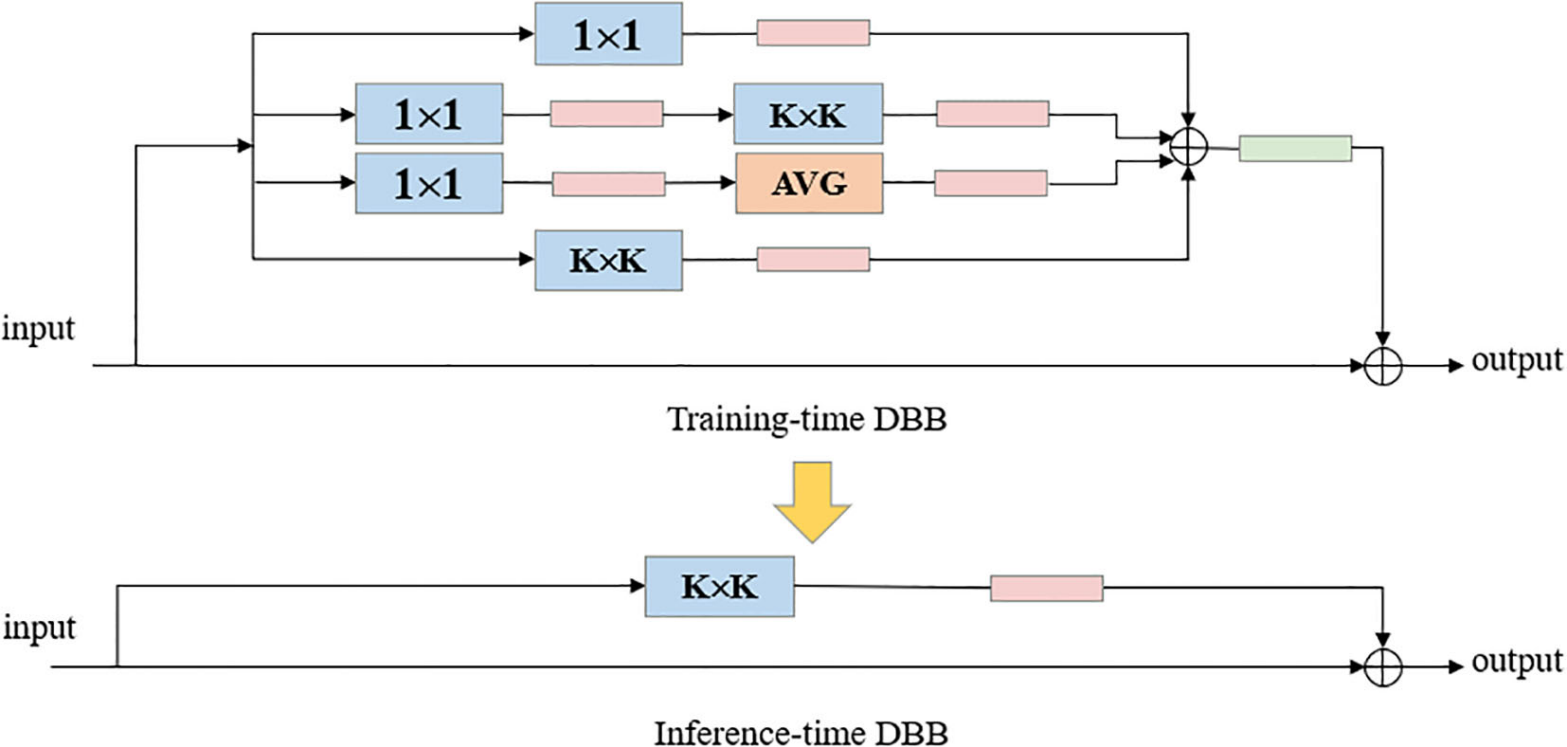
Diverse Branch Block (DBB) structure diagram.

The Diverse Branch Block (DBB) (Ding et al., 2021) adopts an innovative design, replacing Conv in the Bottleneck of C2f with DBB, introducing a multi-branch structure with different receptive fields and complexities, significantly enhancing the detection accuracy of the original model. The DBB design, inspired by the inception architecture, combines multi-scale convolution, sequential 1 × 1—K × K convolution, average pooling, and branch addition multi-branch topology, and the DBB architecture is illustrated in **Figure 5**.

**Figure 6:**
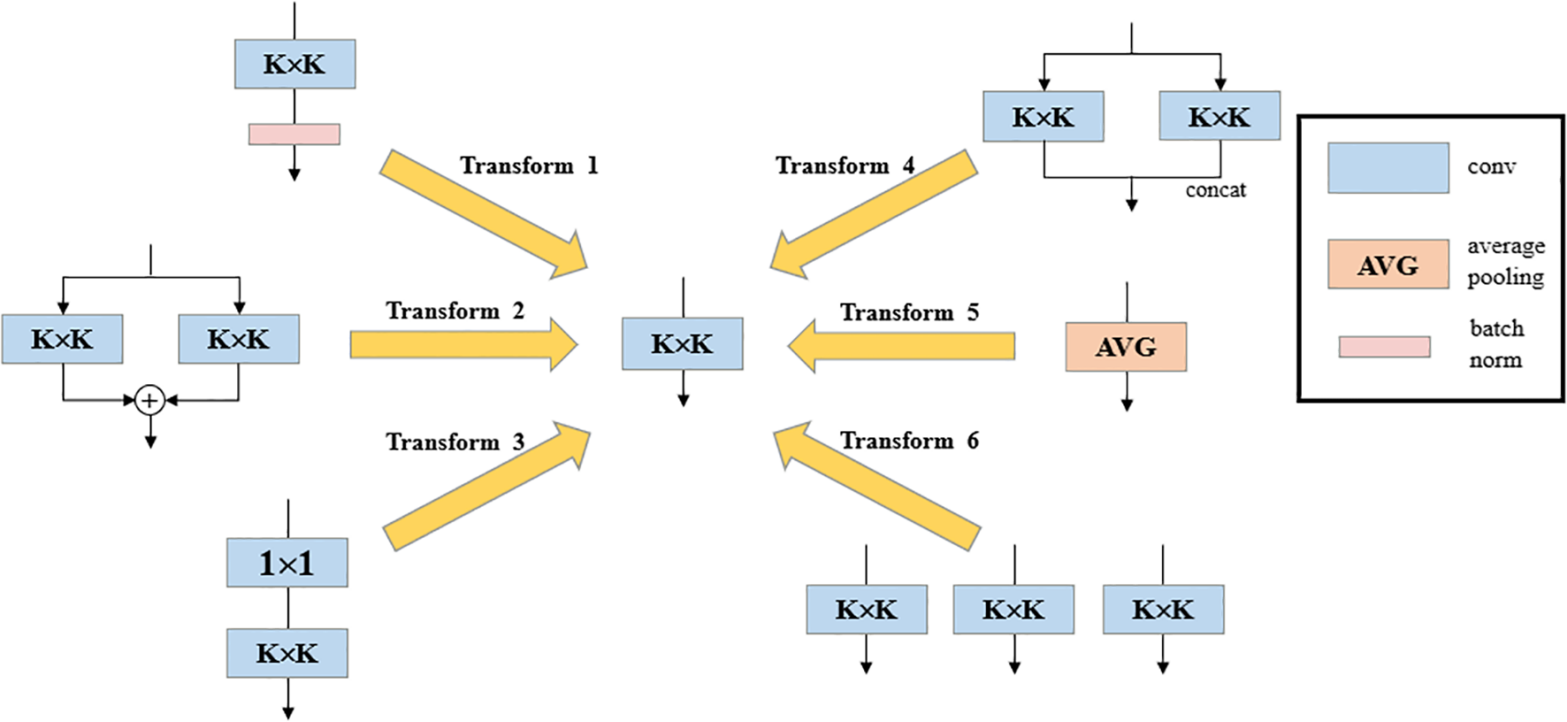
Six transformations to implement an inference-time DBB by a regular convolutional layer.

The DBB module serves as a general-purpose convolutional neural network module that can improve accuracy without loss of inference time. DBB has six different structural transformation methods, Batch Normalization, Branch Addition, Deep Cascade, Multi-Scale Operation, Average Pooling and Convolutional Sequence. The DBB can be converted back to K × K convolution (**Figure 6**) by the six structure-heavy parameter transformation methods, thus improving the detection accuracy without increasing the model complexity and computation, and without worrying about the cost of additional inference time. Compared with the original YOLO v8-Seg backbone network constructed based on the C2f feature extraction module, the backbone network designed in this paper is able to achieve a considerable feature extraction capability with a smaller number of parameters.

#### 2.3.5 Salt tolerance evaluation algorithm for wild rice

By using image acquisition equipment, clear and comprehensive leaf images are obtained. Image segmentation technology is used to separate leaves from complex backgrounds and accurately distinguish different state regions such as green leaves and yellow leaves. The degree of leaf damage data obtained through analysis is used to output the evaluation results of leaf salt tolerance, providing key technical support for the breeding of salt tolerant plant varieties, agricultural production in saline alkali land, and ecological research. The specific calculation steps are as follows:

1. Divide the green and yellow leaves of wild rice.
2. Calculate the number of pixels for green and yellow leaves based on the segmentation results.
3. Calculate the ratio of yellow leaf pixels to total leaf pixels as the basis for grading salt tolerance in wild rice. The calculation formula is shown in Equation 1:

(1) $$P=\frac{C_{\text{Leaf death }}}{C_{\text{Leaf}}}$$

$C_{\text{Leaf death }}$ representing the area of the segmentation leaf death, $C_{\text{Leaf}}$ representing the total area of segmentation blades.

4. Estimate the salt tolerance level and severity of salt stress on wild rice leaves based on the proportion of leaf death area to total leaf area.

### 2.4 Evaluation indicators for salt tolerance of wild rice

Salt tolerant phenotypes of wild rice are usually characterized by growth and development indicators such as high seed germination and seedling survival, low leaf mortality and high yield under salt stress conditions. These phenotypic characteristics reflect the adaptability and tolerance of wild rice to salt stress. The collected data were investigated, including the damage situation, determination of leaf death rate, survival rate, and grading evaluation, and the evaluation criteria were adopted from ‘Identification and Evaluation of Drought- and Salt Tolerant Resources of Hainan Common Wild Rice’ (Tang et al., 2016), as shown in **Table 1**.

**Table 1:** Criteria for the evaluation of salt tolerance in wild rice seedlings.

Grade	Salt Damage Symptoms	Leaf death rate(%)	Salt tolerance
1	Leaves, growth, tillering normal	⩽10	Very strong
3	Growth, tillering inhibited, few leaves curled	<10~25	Strong
5	Growth and tillering severely inhibited, most leaves curled	<25~50	Medium
7	Growth and tillering stopped, most leaves greened up, some plants died	<50~80	Weak
9	Almost all leaves are green or dead	>80	Very weak

Leaf death rate, survival rate and number of green leaves per plant were counted at the end of the experiment for calculation (Song et al., 2014).

Leaf death rate: Leaves showing yellow leaf were counted and leaf death area was calculated. Leaf death rate (%) = leaf death area ÷ total leaf area of test plant × 100

Survival rate: The whole plant without green leaves was recorded as the death of the plant, and the number of surviving plants after the experiment was recorded. Survival rate (%) = number of surviving plants ÷ total number of plants × 100.

The number of green leaves of single plant: the area of green leaves of leaf blades under salt stress was recorded as 1 piece if the area of green leaves of leaf blades under salt stress was more than 1/2, and vice versa was recorded as 0. The number of green leaves of single plant = total number of green leaves ÷ number of surviving plants. Similarly, the number of green leaves per plant under non-salt stress was counted.

## 3 Experimental results and analysis

### 3.1 Indicators for model evaluation

The configuration of the operating environment for this experiment included an operating system environment of Windows 11, a processor of 12th Gen Intel(R) Core(TM) i5-12500 3.00 GHz, 32G of machine-banded operating memory, a 1TB SSD, and a graphics card of NVDIA GeForce RTX 3080 with 10GB of video memory using GPU-accelerated computing. Software environment: Python 3.9, PyTorch 1.7.0, Torchvision 0.8.2, CUDA 11.0, as shown in **Table 2**. The number of trial iterations was set to 800, batch-size was set to 8, and Adam was used as the optimizer. The initial learning rate of the model was set to 1e-3, the maximum learning rate was 1e-5, momentum was 0.937, weight decay was 0, and the input image resolution was set to 640oluti the same training parameters and dataset were used for all models during the training process.

**Table 2:** The configuration of the test environment.

Mark	Parameter
Operating system	Windows11
CPU	12th Gen Intel(R) Core(TM) i5-12500
GPU	NVIDIA GeForce RTX 3080
Video storage	10GB
GPU Acceleration Library	CUDA11.0, CUDNN v8.0.5
Training framework	PyTorch
Programming Languages	Python3.9
Compilation environment	Pycharm

The performance of the model was evaluated using precision P(precision), R(recall), F1-score, $mAP_{@0.5}$ (mean average precision) and computational costs (FLOPS) (Huang et al., 2021) (Pietrołaj and Blok, 2022). As shown in Equations 2–Equation 6.

Precision is the ratio of correctly predicted positive samples to all predicted positive samples, and is calculated as follows:


(2) $$\text{Precision}=\frac{\text{TP}}{\text{TP}+\text{FP}}$$

where TP denotes true positives and FP denotes false positives.

Recall measures the proportion of actual positive samples correctly identified by the model, calculated as follows:


(3) $$\text{Recall}=\frac{\text{TP}}{\text{TP}+\text{FN}}$$

where FN represents false negatives.

The F1 metric is the reconciled mean of accuracy and recall, which serves to combine accuracy and recall. The calculation formula such as the Equation 4:


(4) $$\text{F}1=2\cdot \frac{\text{Precision}\cdot \text{Recall}}{\text{Precision}+\text{Recall}}$$

$mAP_{@0.5}$ (mean average precision) is calculated from a precision-recall curve and defined as follows:


(5) $$mAP_{@0.5}=\frac{\sum_{i=1}^{N}APi}{N}$$

Here, $mAP_{@0.5}$ refers to the average AP when the Intersection over Union (loU)threshold is 0.5.

FLOPS (Floating-Point Operations Per Second) measures the computational costs, and is calculated as follows:


(6) $$FLOPs=\sum^{}\left(K\times K\times C_{in}\times C_{out}\times H\times W\right)$$

Where H×W is the size of the output feature map

### 3.2 Ablation and comparison experiments

#### 3.2.1 Ablation experiment

Ablation experiments were performed on the constructed dataset and the results are shown in **Table 3**. YOLOv8-Seg was used as the baseline model. Compare with the following improved models respectively: as shown in the table, adding the CAFM attention mechanism in YOLOv8-Seg, P, R, F1 and $mAP_{@0.5}$ are all increased respectively; when DBB replaces Conv at the bottleneck of C2f in the YOLOv8-Seg model, P decreases by 0.3%, but R, F1 and $mAP_{@0.5}$ are relatively improved, and DBB can enrich the feature space, provide fields and paths with different complexity feelings, and enhance the feature extraction ability of the model. Adding the two together to get the final model, its P, R, F1 and $mAP_{@0.5}$ increase effect is obvious, respectively, 92.8%, 87%, 89.81% and 90.9%, the improvement of the model is improved due to other models.

**Table 3:** Experimental results of model ablation.

Group	Model	P/%	R/%	F1/%	/%	FLOPs
1	YOLOv8-Seg	89.3	82.1	85.55	88.2	16.5
2	+CAFM	91	83.1	86.87	88.6	13.2
3	+C2f_DBB	89	83.6	86.22	88.9	9.6
4	+C2f_DBB+CAFM	92.8	87	89.81	90.9	8.7

#### 3.2.2 Comparison experiment

**Figure 7:**
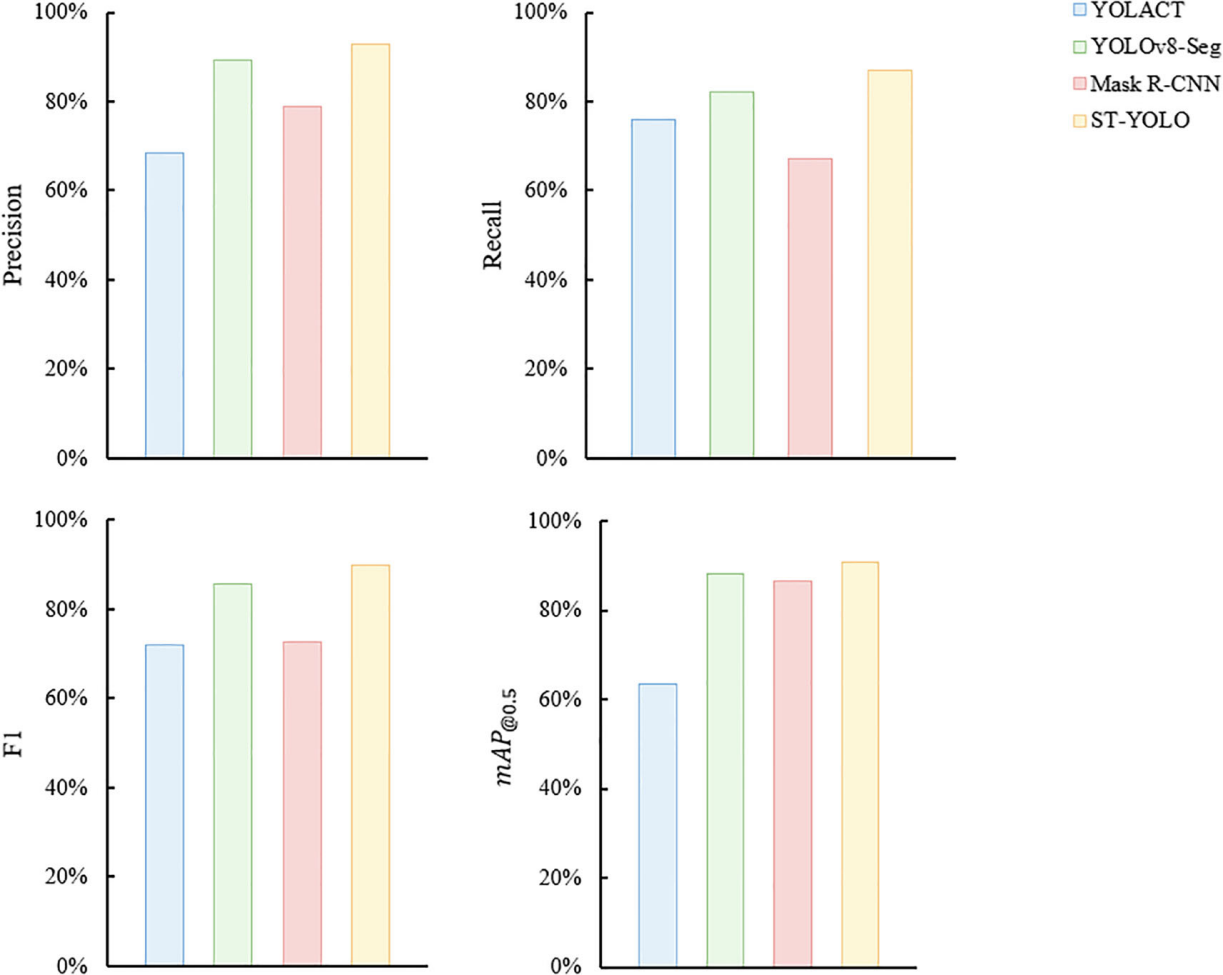
Comparison of detection performance for different networks.

In order to evaluate the ST-YOLO model proposed in this paper more comprehensively, the performance is compared with example segmentation models such as YOLACT, Mask R-CNN and YOLOv8-Seg. The same dataset is used for all experiments, which consists of 1422 training images, 406 validation images and 204 test images. We maintained the same experimental conditions throughout the experiments to ensure the fairness of the comparison. The comparison results are shown in **Figure 7**, which shows that ST-YOLO performs better in P, R, F1 and $mAP_{@0.5}$ , and significantly outperforms YOLACT, Mask R-CNN and YOLOv8-Seg.

**Figure 8:**
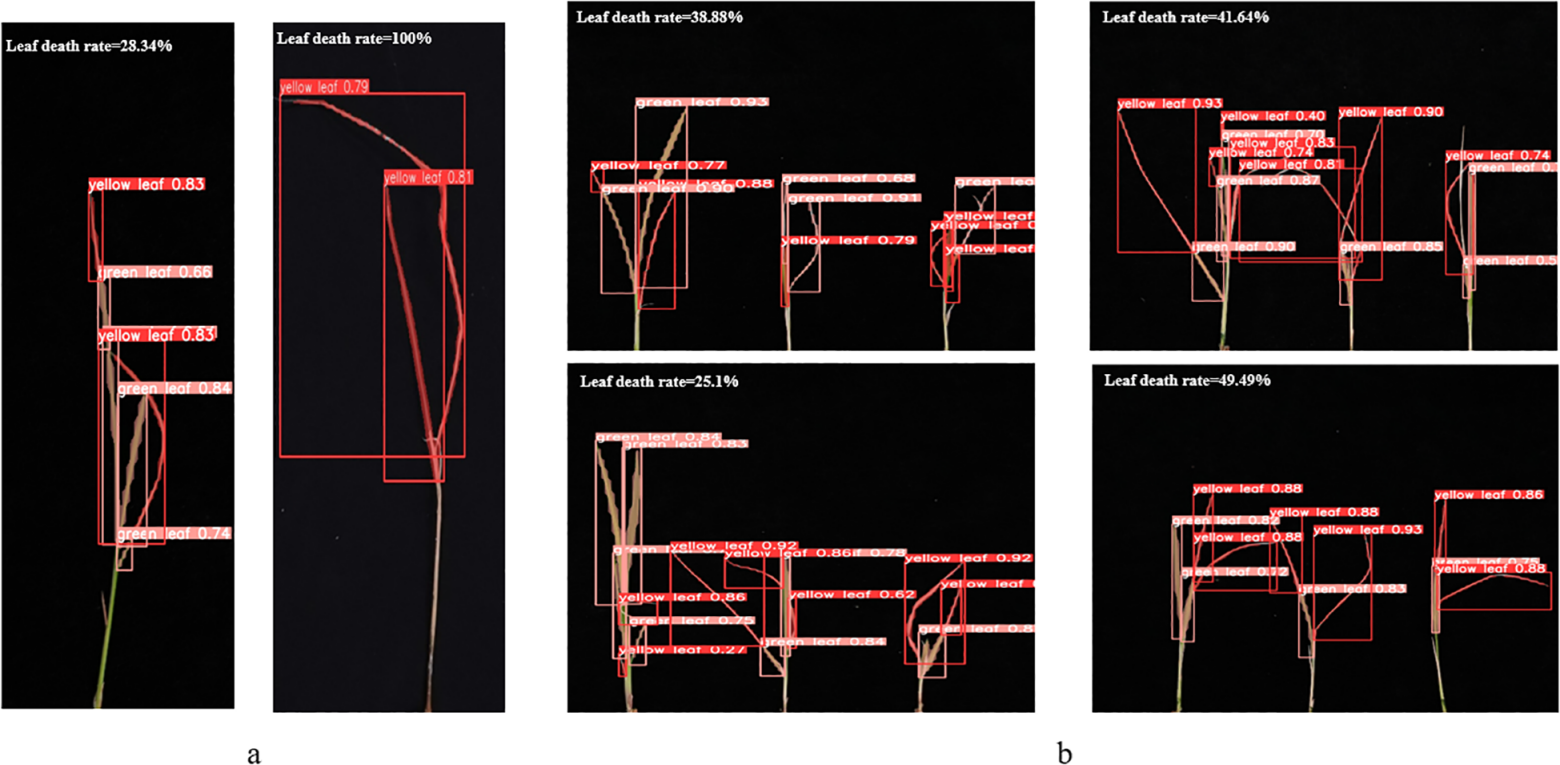
ST-YOLO detection results. **(a)** Results of single-strain testing. **(b)** Results of multi-strain testing.

As shown in **Figure 8**, **Figure 8a** demonstrates the results of single-plant detection, while **Figure 8b** demonstrates the results of multiple-plant co-detection performed by ST-YOLO. The ST-YOLO model was used to collect leaf death rate data for the identification of salt tolerant phenotypes evaluated at the seedling stage of wild rice. The introduction of the C2f-DBB into ST-YOLO achieves a reduction of the model size and the computational cost, while maintaining a comparable segmentation accuracy, the It is able to obtain a large feature extraction capability with a small number of parameters; CAFM can extract features at different scales by designing a multi-scale feed-forward network, which improves the detection performance for small targets.

#### 3.2.3 Comparative experiment on evaluation indicators

In order to comprehensively assess the effectiveness of the proposed ST-YOLO model for practical applications, a comparative study with manual testing in the time dimension was carried out. The same dataset was utilized for all experiments, and in the model performance evaluation session, a systematic test was conducted for the salt tolerance evaluation effectiveness of the ST-YOLO model. The experimental results demonstrate that the evaluation outcomes of the ST-YOLO model exhibit a high degree of consistency with the manual expert evaluation, with a consistency coefficient exceeding 90%. In terms of time efficiency, high-precision timing equipment was employed to synchronize the measurement of the model and manual evaluation. The single image processing time of the ST-YOLO model was 1.2 seconds, while the average time consumed by professionals under the same task conditions was 100 seconds. The processing efficiency of the model was improved by 98.2% compared with that of the manual evaluation, and the specific results are shown in **Table 4**. This outcome unequivocally validates the ST-YOLO model’s substantial time efficiency advantage while maintaining a commendable level of accuracy.

**Table 4:** Comparison of evaluation indicators.

Method	Testing index	Time/s
Manual testing	Leaf death rate	100
ST-YOLO	Leaf death rate	1.2

### 3.3 Model test experiment for the identification of wild rice phenotypic evaluation of salt tolerance

**Figure 9:**
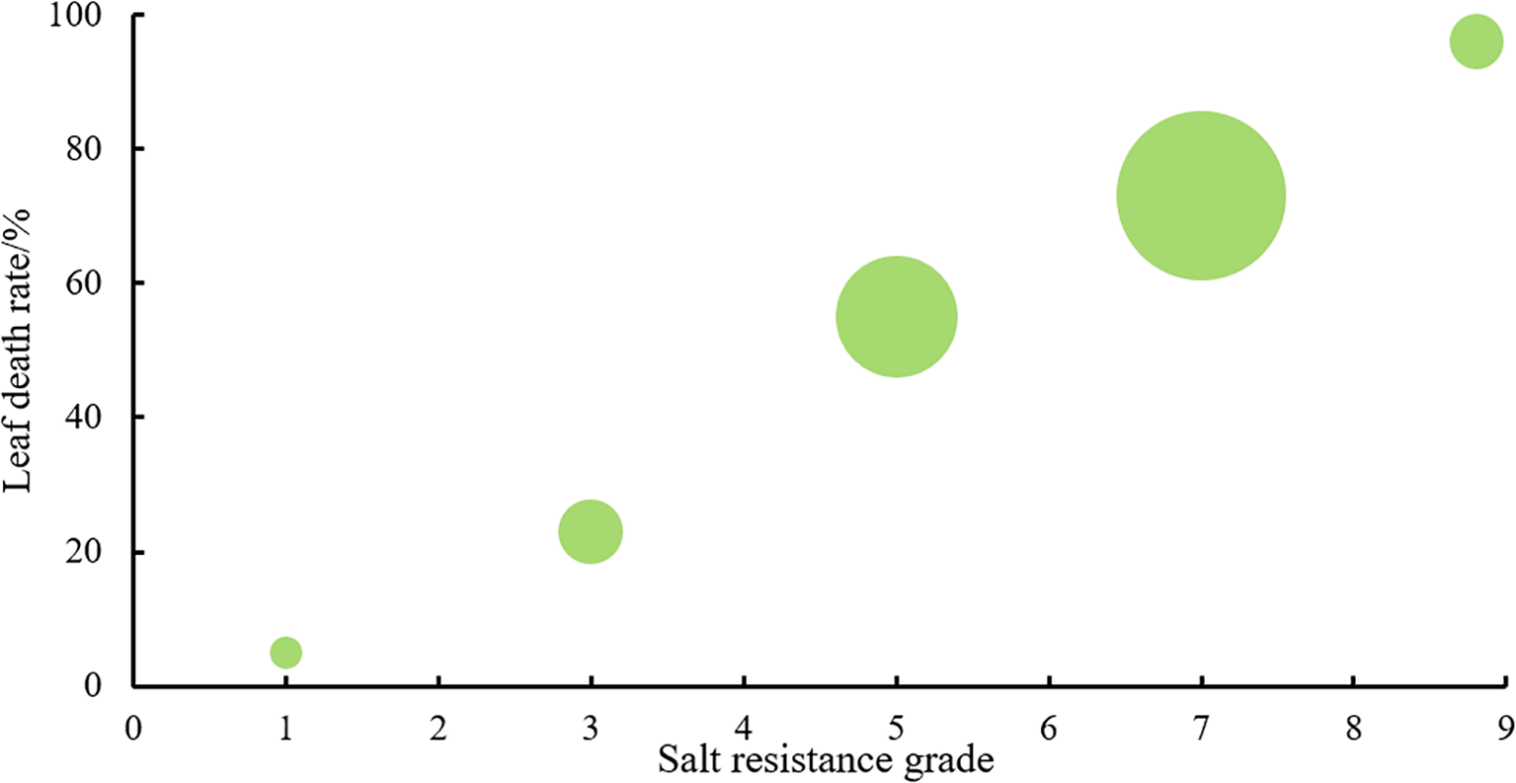
Distribution of salt damage in wild rice leaves.

A deep learning ST-YOLO model was employed to identify the salt tolerance phenotype evaluation of wild rice 885-1095. The dataset was classified according to its salt tolerance grade, and the specific data on the percentage of yellow leaves observed under different salt tolerance grades are presented in **Figure 9**.

**Figure 10:**
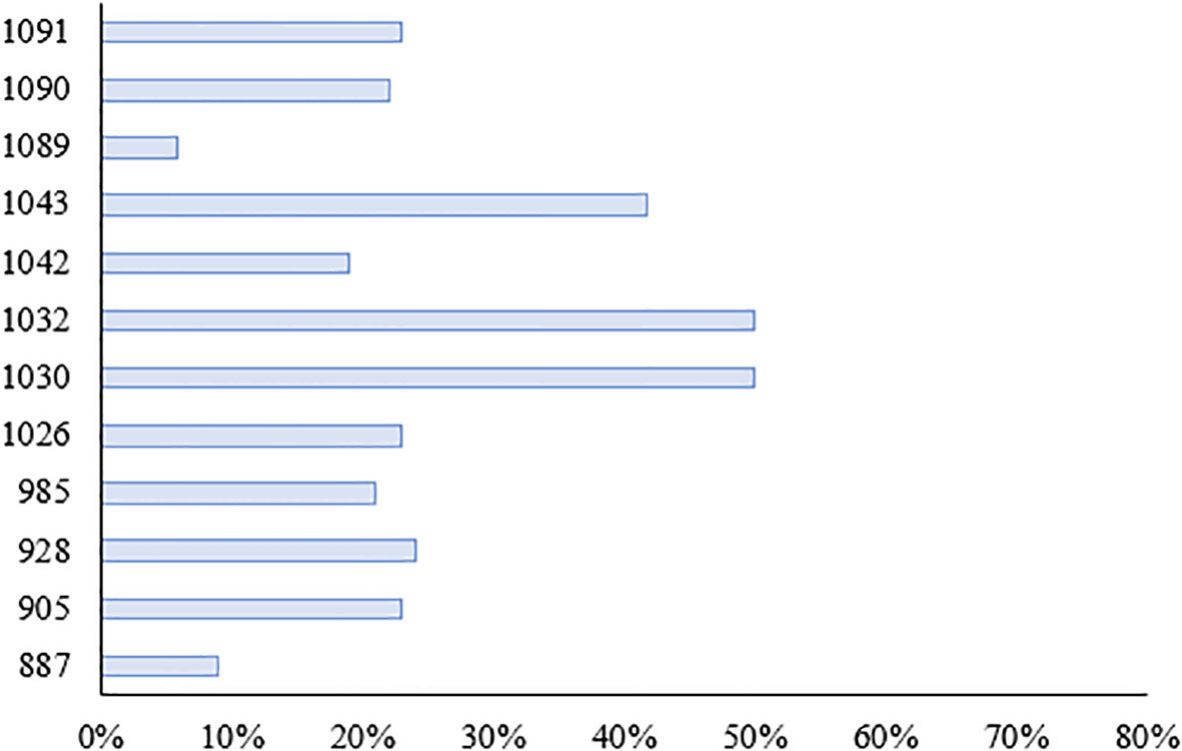
Percentage of leaf death rate in salt stress varieties.

**Figure 11:**
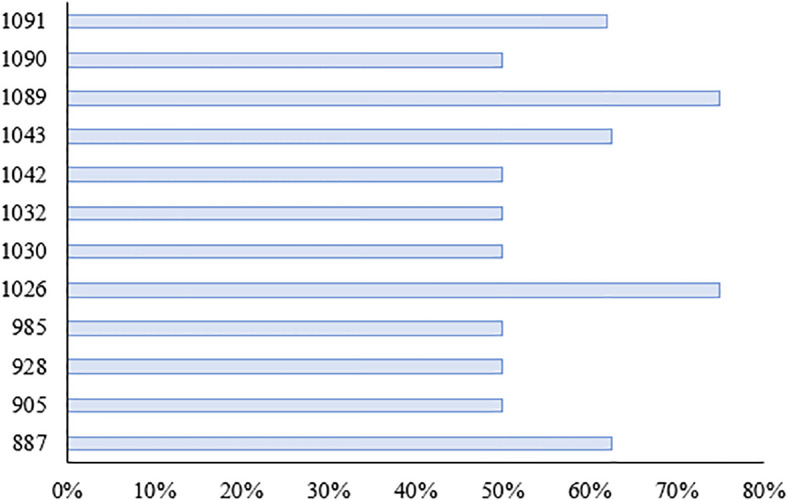
Survival rate of each species under salt stress.

We investigated the seedling leaf death rate, survival rate, number of green leaves per plant and salt tolerance grade. Under salt stress, the survival rate and the number of green leaves per plant generally showed a decreasing trend, with larger values indicating that the variety was more salt tolerant; while the leaf death rate and salt tolerance grade generally showed an increasing trend, with larger values indicating that the variety was less salt tolerant. Changes in these indicators can help assess the performance of salt tolerance in wild rice under salt stress conditions. From **Figures 10**, **11** and **Table 5**, it can be observed that the leaf death rate and survival rate of each participating variety were less than 1. In terms of leaf death rate, varieties 887 and 1089 had the smallest values and performed the best, while in terms of survival rate, most of the varieties were around 60%, with 1026 and 1089 performing the best, with a survival rate of 75%. According to the number of green leaves per plant, varieties 887, 1030 and 1032 performed the best, with more than three leaves per plant. Among them, varieties 887 and 1089 had a salt tolerance rating of 1, which is very high; 1030, 1032 and 1043 had a salt tolerance rating of 5, which is medium, while the other varieties had a salt tolerance rating of 3, which is high. It was able to assess the performance of the varieties in terms of salt tolerance under salt stress conditions.

**Table 5:** Morphological indicators of each restoration line under salt stress.

Numbers	The number of green leaves of single plant	Salt Resistance Grade
887	3.3	1
905	2.0	3
928	1.5	3
985	2.5	3
1026	2.8	3
1030	3.0	5
1032	3.0	5
1042	2.3	3
1043	2.5	5
1089	2.8	1
1090	2.8	3
1091	2.8	3

### 3.4 Classification of salt tolerance grades in wild rice

The salt tolerance evaluation of the ST-YOLO model was used to compare with the manual salt tolerance evaluation in order to calculate the evaluation accuracy of the model. According to the wild rice seedling salinity tolerance evaluation criteria divided into five salinity tolerance levels, in order to further verify the performance of the ST-YOLO model, different salinity tolerance was evaluated, as shown in **Table 6**.

**Table 6:** Assessment of different salt tolerance grades at seedling stage in wild rice.

Original Image	Dead Leaf Area/cm^2^	Leaf Area/cm^2^	Leaf Death Rate/%	ST-YOLO	Salt Resistance Grade
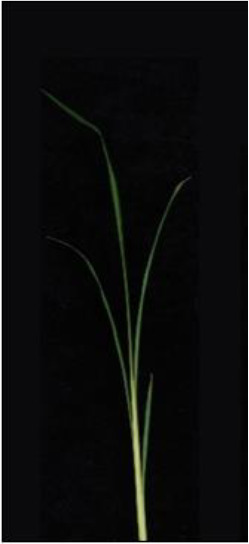	1.13	17.4	6.52	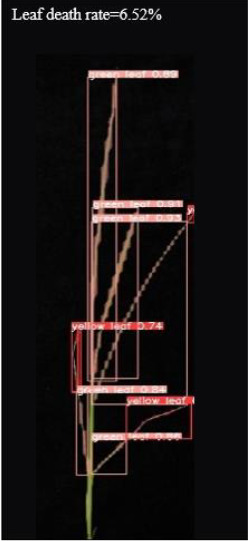	1
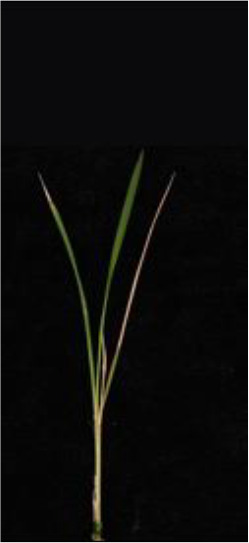	2.50	15.5	16.11	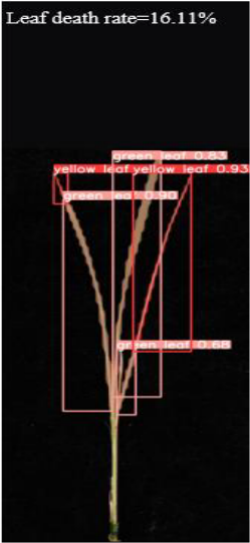	3
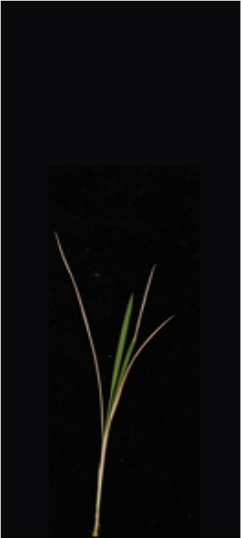	6.33	13.9	45.57	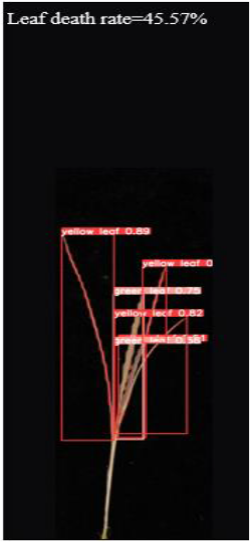	5
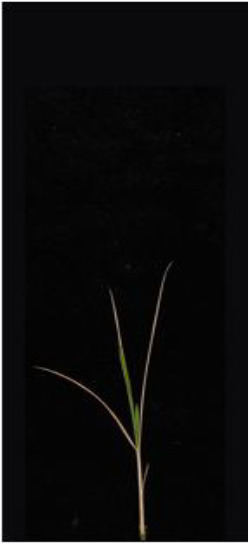	4.50	8.3	54.27	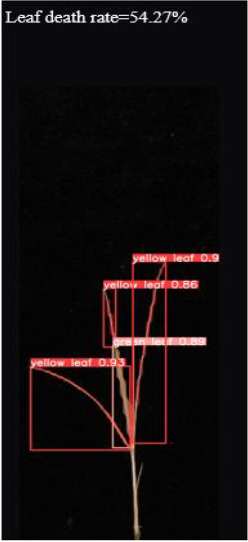	7
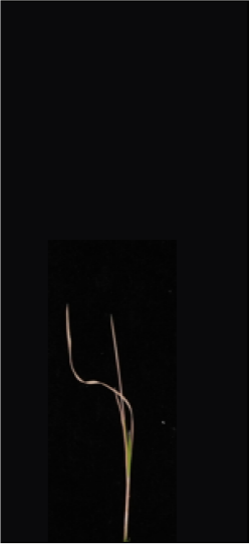	4.78	5.8	82.43	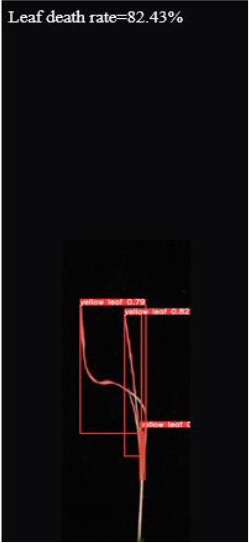	9

The accuracy of salt tolerance evaluation of ST-YOLO model reached 90.9%. The systematic analysis of wild rice leaves with different salt tolerance through the model can evaluate the changes in the growth status and morphological characteristics of leaves under salt stress, and ST-YOLO showed stable evaluation performance in wild rice materials with different salt tolerance.

**Figure 12:**
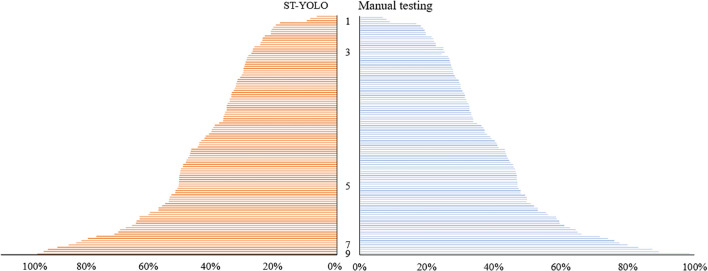
Distribution of salt treated plant height to control length ratio at seedling stage of wild rice germplasm.

The salt tolerance grades of the test set were determined manually and with the ST-YOLO model, and the results were compared with those obtained with manual detection, as illustrated in **Figure 12**. The accuracy and efficiency of the ST-YOLO model in determining salt tolerance grades were evaluated, thereby providing a more comprehensive reference basis for assessing the salt tolerance of wild rice. The percentage of salt tolerant leaf death rate of wild rice leaves, as determined by the ST-YOLO model, can be used to classify the salt tolerance grade of wild rice. By accurately measuring and analyzing the percentage of leaf death, the ST-YOLO model can accurately assess the leaf health of wild rice under salt stress conditions, thereby providing an important basis for the assessment of salt tolerance capacity. This method of classifying salt tolerance grades based on data and proportions helps to provide a more comprehensive understanding of the performance of wild rice in coping with salt stress, and provides strong support for further research and improvement of salt tolerant varieties.

### 3.5 Development of a salt tolerance phenotype evaluation system for wild rice seedlings during the seedling stage

**Figure 13:**
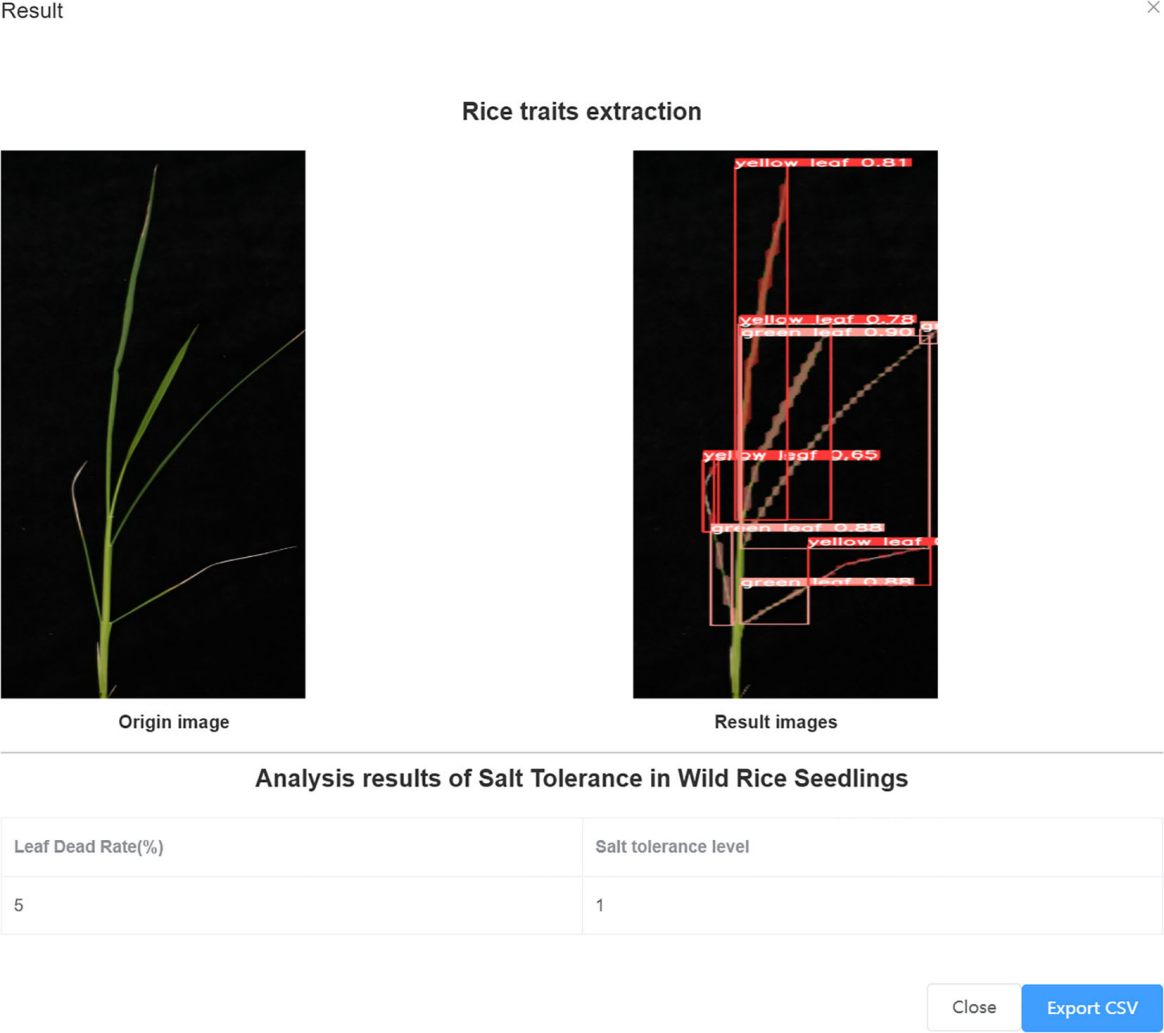
Results of salt tolerance identification at seedling stage of wild rice.

The system is designed and developed using Pycharm and PyQt, combined with usage scenarios and user needs. The appearance interface design focuses on simplicity, with two buttons designed for image selection and recognition detection. Select the image and click the recognition detection button to obtain the recognition result, leaf death rate, and salt tolerance level of the image at the bottom of the interface. As shown in **Figure 13**.

The salt tolerance phenotype evaluation system for wild rice seedlings adopts a web-based interactive design, supporting remote operation and data management for multiple users and roles, and achieving integrated services from data collection to cloud based intelligent analysis. The data-driven phenotype detection algorithm carried by the system provides precise and efficient technical support for modern agricultural breeding research.

## 4 Discussion and conclusions

The application of deep learning technology can also improve the efficiency and accuracy of salt tolerance evaluation. Traditional salt tolerance evaluation usually takes a lot of time and manpower, while deep learning models can achieve automated detection and evaluation, greatly improving work efficiency. Meanwhile, the accuracy of the deep learning model can more accurately evaluate the salt tolerance of different wild rice varieties, which provides an important reference for the subsequent breeding work. This study aims to meet the practical application needs of wild rice salt tolerance detection, and proposes an innovative ST-YOLO wild rice salt tolerance detection method, which includes C2f improvement based on the DBB module and the addition of CAFM attention mechanism after backbone and before detect. The main conclusions are as follows:

1. By performing DBB improvement on the C2f structure, we successfully enhance the learning capability of a single convolutional layer, which not only improves the accuracy without increasing the number of parameters and computation, but also reduces the inference time and processing time. This improvement not only enhances the information fusion capability of Neck, but also significantly improves the overall accuracy. Meanwhile, the CAFM attention mechanism, which does not increase the original network parameters, is introduced to further improve the detection accuracy and speed.
2. The experimental results show that our proposed ST-YOLO model achieves an impressive result of 90.9% in $mAP_{@0.5}$ . Meanwhile, P and R reached 92.8% and 87%, respectively. Compared with the original model, $mAP_{@0.5}$ improves by 2.7%, showing a significant performance improvement. These results not only prove the effectiveness of the ST-YOLO model, but also show that it outperforms other current algorithms in terms of the performance of $mAP_{@0.5}$ , P and R.
3. Salt tolerance rating of 286 varieties was assessed by the ST-YOLO model, and wild rice salt tolerance rating was performed by calculating the leaf death rate of the varieties, and a total of two varieties with very strong salt tolerance and seven varieties with strong salt tolerance were screened out. Although the ST-YOLO model met the requirements of real-time and accuracy in the wild rice leaf salt tolerance detection task, there were still some leakage and misdetection in the practical application. Future research efforts will focus on improving the anchor frame generation strategy and the method of eliminating overlapping detection frames to further reduce the leakage and false detection rates and enhance the stability and reliability of the model. These efforts will help to further promote the development and application of salt tolerance detection technology in wild rice.

## Data Availability

The original contributions presented in the study are included in the article/supplementary material. Further inquiries can be directed to the corresponding author.
